# Data quality and associated factors in the health management information system at health centers in Shashogo district, Hadiya zone, southern Ethiopia, 2021

**DOI:** 10.1186/s12911-022-01898-3

**Published:** 2022-06-15

**Authors:** Nigusu Getachew, Bereket Erkalo, Muluneh Getachew Garedew

**Affiliations:** Department of Health Policy and Management, Faculty of Public Health, Health Institute, Jimma University, P.O. Box 378, Jimma, Ethiopia

**Keywords:** Data quality, Health management information system, Shashogo district, Health centers

## Abstract

**Background:**

Poor quality routine data contributes to poor decision-making, inefficient resource allocation, loss of confidence in the health system, and may threaten the validity of impact evaluations. For several reasons in most developing countries, the routine health information systems in those countries are described as ineffective. Hence, the aim of this study is to determine the quality of data and associated factors in the routine health management information system in health centers of Shashogo district, Hadiya Zone.

**Methods:**

A facility-based cross-sectional study was conducted from June 1, 2021, to July 1, 2021, and 300 participants were involved in the study through simple random sampling. The data was collected with a self-administered questionnaire by trained data collectors. After checking its completeness, the data was entered into EPI data version 3.1 and exported to SPSS version 25 for statistical analysis. Finally, variables with *p* < 0.05 during multivariable analysis were considered significant variables.

**Result:**

A total of 300(100%) participant were included in the interview and HMIS data quality was 83% in Shashogo district health centers. The data quality in terms of accuracy, completeness, and timeliness was 79%, 86%, and 84%, respectively. Conducting supportive supervision [AOR 3.5 (1.4, 8.9)], checking accuracy [AOR 1.3 (1.5, 3.5)], filling registrations [AOR 2.7 (1.44, 7.7)], and confidence level [AOR 1.9 (1.55, 3.35)] were all rated positively found to be factors associated with data quality.

**Conclusion:**

The overall level of data quality in Shashogo district health centers was found to be below the national expectation level. All dimensions of data quality in the district were below 90% in data accuracy, content completeness, and timeliness of data. Conducting supportive supervision, checking accuracy, filling registrations and confidence level were found to be factors associated with data quality. Hence, all stakeholders should give all necessary support to improve data quality in routine health information systems to truly attain the goal of providing good quality data for the decision-making process by considering the identified factors.

## Background

Any system that takes, saves, maintains, or communicates information on people's health or the activities of organizations in the health sector is referred to as a health information system (HIS). Overall, a well-functioning HIS is a coordinated effort to collect, process, report, and use health data and knowledge to influence policy and decision-making, program implementation, individual and public health outcomes, and research [1].

RHIS (routine health information systems) have been used for over a century around the world. Recently, developing countries have begun to place a greater emphasis on RHIS. RHIS has recently gained prominence in developing countries. In 2008, Ethiopia began using the Health Management Information System (HMIS), which is meant to generate routine data for decision-making at various levels of the health system [2, 3].

Data quality in public health has different definitions from different perspectives. These include: fit for use in the context of data users’ timely and reliable data essential for public health core functions at all levels of government; and accurate, reliable, valid, and trusted data in integrated public health informatics networks. Data is the starting point for health care. Information, whether maintained manually or electronically at a large teaching hospital, health center, or health posts, [4–6].

In low- and middle-income countries, health information systems are plagued by inadequate data analysis and poor utilization of routine data for decision-making. According to a Ugandan report, health care professionals who were not trained in computer software, data management, or HMIS were unable to comprehend standard indicators and data quality, resulting in restricted data collection [7].

Despite the fact that routine health data is often inaccessible, under-utilized, incomplete, and not used for institutional decision-making in sub-Saharan Africa, the quality of HMIS in low and middle-income countries is poor, with direct and indirect consequences [8, 9].

Poor quality routine data and data that are not sufficiently timely, credible, accurate, and complete will contribute to poor decision-making, negatively impact strategic planning, inefficient resource allocation, and loss of confidence in the health system, and may threaten the validity of impact evaluations. Access to complete and comprehensive data to guide resource allocation and program improvement efforts is increasingly important given the high burden of disease and limited resources in numerous low- and middle-income countries [2, 10, 11].

A good-quality routine health information system is key to the success of the health information system and the entire health system. High quality data is required to enable safe and reliable health care delivery, and health facility data is a key input to monitoring performance [12, 13]. Most of the studies were conducted about only determinants of data utilization in different places and times, but they did not conduct studies on data quality levels and associated factors. Also, the study was not conducted in Shashogo district concerning data quality and factors that affect the quality of data. Therefore, this study will help to fill this gap in the study areas and identify the factors that affect data quality in Shashogo district, as well as generate or form good quality data in routine health information systems for the use of information and decision-making and planning.

## Method

### Study setting, period and design

A facility-based cross-sectional study design was carried out from June 1 to July 1, 2021 in Hadiya Zone, Shashogo district, found in the Southern Nations, Nationalities, and Peoples Regional State of Ethiopia. The district has a total of 36 Kebele (2 urban and 34 rural), which are located 54 KM away from Hosanna town. The district has a total population of 145,244 (72,912 females and 72,332 males). There are five health centers, twenty-eight clinics, and seven drug stores. The district has 342 health professionals in different types of departments.

The source and study population were all health centers in the districts and sampled functional health centers and health workers who were working in the routine HIS. Health facility assessments can be implemented as a census for document review [14], according to the HMIS information use guide line of HMIS using all health centers in the Woreda to conduct RDQA and the national acceptable range of RDQA in administration units. The levels are 90–110%[3]. And LQAS provides a quick and reliable method for comparing compiled, recorded, and reported data. The data should correspond with LQAS results above 90% [15].

### Sample size and sampling technique

A single population proportion formula was used to calculate sample size, which was based on the following: Assumption: 75% of people are capable of performing HIS tasks in Eastern Ethiopia [16]. The desired degree of precision was 5%, with a 95% confidence interval. This results in a sample size of 286 and, using a contingency of 5% for non-respondents, the final sample size will be 300. For all five health centers, which are found in the district, and for respondents of the self-administered questionnaire, the number was proportionally allocated to each health center beyond the numbers. Those are Bonosha, Doesha, Jemeya, Hiriko, and Shamo health centers, and the numbers in the health centers are 99, 56, 75, 49, and 43, respectively. The FMOH guideline of HMIS says that all health professionals who are involved in HMIS activities, starting from the daily register of the source document to the final report, are included [3]. Health professionals for the self-administered questionnaire were selected by using simple random.

### Data collection instrument and technique

Data collection tools were developed from the HMIS user’s guideline and PRISM assessment tools [17]. The tool is prepared to fit with the local context, and it mainly contains questions to assess the accuracy, completeness, and timeliness of HMIS data and a self-administered structured questionnaire containing background information on the respondent’s organizational, behavioral, and technical determinants of data quality in health centers. Data was collected by four health officers who were recruited based on their experience and trained on HMIS related tasks. For those selected data collectors, training was given on the questionnaires, data collection methods, and procedures for the next two days. The data collectors were to collect the data from each respondent and review the document or registration for data quality assessments. The questionnaires were adapted from standard tools and then translated into Amharic. The tool was pretested prior to the actual data collection period on 5% of the sampled health professionals. During the data collection period, supervision of data collection procedures was conducted by the principal investigator and onsite technical assistance was given to data collectors.

### Operational definition

HMIS Data quality:—was measured by calculating the sum of three dimensions of data quality measured and dividing by for those three dimensions, then taking the average’s scores.

Data accuracy:-was measured by calculating the number from source document or register divided by the number from report submitted to the next level. Based on 10% tolerance for data accuracy was classified:-Over reporting (< 0.90 or 90%), Acceptable limit (0.90–1.10 or 90–110%) and under reporting (> 1.10 or 110%).

Content completeness:-was measured by the number of cells of report form which are left blank without indicating “zero”. If greater than or equal to 90% of cells of the report filled was considered as complete.

Report timeliness:-was measured by the number of reports delivered up to deadline for facility head divided by the number of reports expected to come.

Data item: –an HMIS indicator that is selected to assess the data accuracy.

### Data processing and analysis

The completed questionnaire was coded and entered into a data entry template in EPI-DATA version 3.1, then exported to SPSS version 25 for analysis. Descriptive statistics like frequencies, percentages, tables, graphs, and charts were employed. In the bi-variable logistic regression analysis, p-values of less than 0.25 were used to select the candidate variables for multivariable logistic regression analysis. An adjusted odds ratio (AOR) with a 95%CI was used to determine the predictor of the outcome variable independently and to show the strength of an association. A p-value of less than 0.5 was considered statistically significant.

## Result

### Socio-demographic related variables of study participants

A total of 300(100%) participant were included in the interview and five health center heads (1.7%), 48 department heads (16%), five HMIS focal persons (1.7%), and 242 health service providers (80.7%) participated in the study. Of the total respondents, 66.7% were male, and 165 (55%) had attended diploma level education. The mean age of respondents in this study was 31. Of all the respondents, about 100 (33.3%) of them were nurses. With the maximum experience of 18 years and a minimum experience of 1 year, (See Table 1).

**Table 1:** Socio demographic characteristics respondents of health centers in Shashogo district 2021 *n* = 300

variables	category	Frequency	Percent
Sex of respondents	Male	200	66.7
	Female	100	33.3
Age of respondents	21–25	26	8.6
	26–30	127	42.3
	31–35	112	37.3
	36–40	33	11
	> 41	2	0.6
Level of education	diploma	165	55
	Bachelor degree	133	44.3
	Master’s degree	2	0.66
Field of study	Nursing	100	33.3
	midwife	55	18.3
	Health officer	69	23
	Lab. technology	33	11
	Health information technology	10	3.3
	Pharmacy	15	5
	Medical Doctors and emergency surgeries	18	6
Position of person interviewed	Head of health center	5	1.7
	Department head	48	16
	HMIS focal	5	1.7
	Service providers	242	80.7
Year of experience	1–5	101	34.3
	6–10	123	41
	11–15	69	23
	> 16	5	1.66

**General structures of HMIS:** Five (100%) health centers have assigned HMIS focal persons who are responsible for reviewing and aggregating numbers prior to submission to the next level, and all health centers are assigned HIT professionals.

**Record keeping:** According to the findings, all 5 (100%) of health centers had kept copies of service delivery reports, which is counted as one report copy in each health center submitted to the district health office in one Ethiopian fixed-year report, and the district's average number of kept report copies was 11 months.

### Data accuracy

**Fig. 1:**
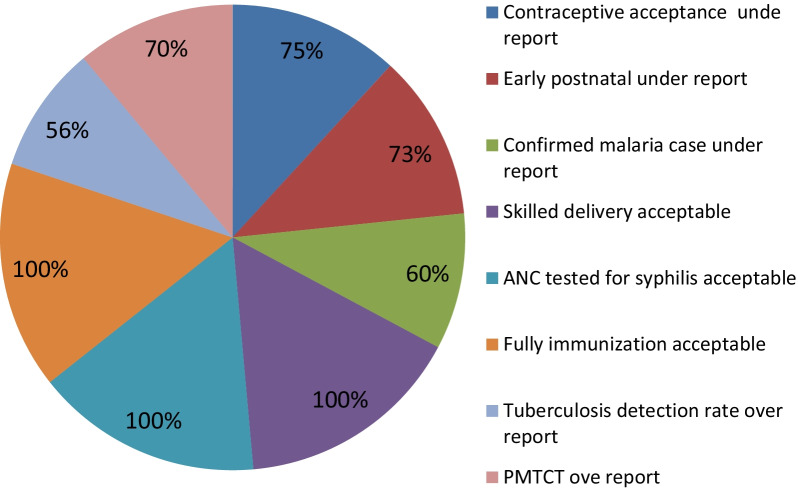
Accuracy level of data in each data item in shashogo district health center 2021

**Fig. 2:**
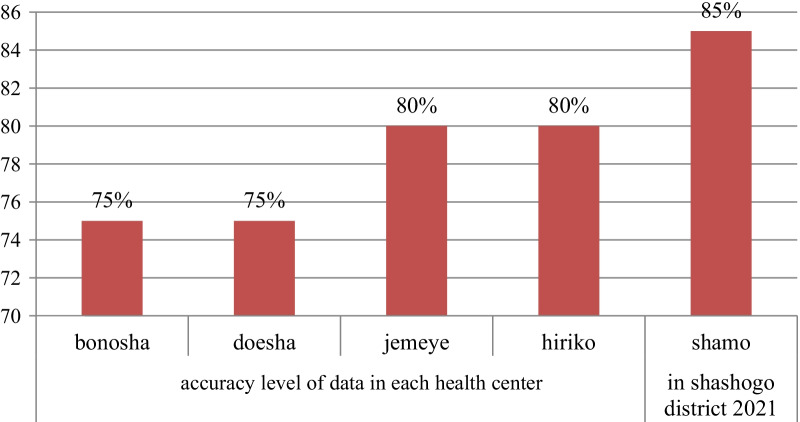
Data accuracy in each health centers in shashogo district health centers 2021

A total of five health centers' data accuracy was total contraceptive accepters (75%), and TB detection service (56%). The data accuracy level was 85% in Shamo health center and 75% in Bonosha health center. The data accuracy at the district level was 79%. (See Figs. 1–2).

### Data timeliness and completeness

**Fig. 3:**
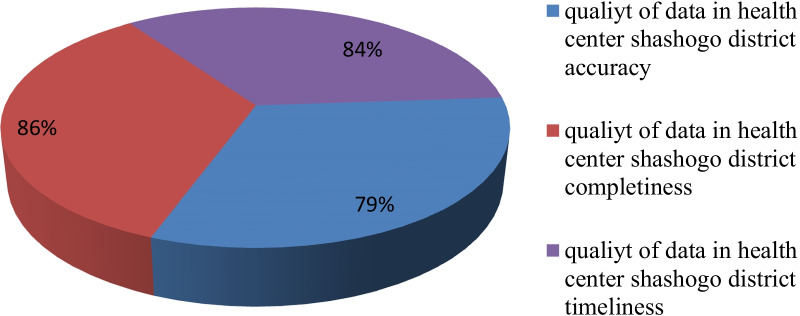
Level of data quality in the shashogo district health center 2021

Content completeness data in the district health center was scored at 86%. The report showed that Bonosha, Doesha, Jemeye, Hiriko, and Shamo health center timeliness of data was 90%, 88%, 85%, 75%, and 83%, respectively. Overall, 84% of the HMIS reports sent were received by the reporting deadline. Depending on the three dimensions of data quality, which are accuracy, completeness, and timeliness, the overall data quality of the district health centers was 83%. (Fig. 3).

### HMIS process

Out of 300 respondents, 196 (65.3%) participate in aggregation or compilation of data from registration, and more than half of the respondents, 195 (65%), reported that they conduct or check the quality of health data.

### Technical factors

Among 300 total respondents, 196 (65.3%) reported that they had a standard set of indicators, including case definitions, in their departments. Sixty-five percent (65%) of the participants reported that there are skilled staff able to aggregate data and to fill out the reporting formats, and of those respondents, about 235 (78%) of professionals reported that HMIS is a user-friendly format, and only 75 (22%) of respondents did not understand the format.

### Behavioral factors

From 300 respondents, about 279 (93%) of respondents got training opportunities towards HMIS, and about 168 (56%) reported on data quality checking skills. (Table 2).

**Table 2:** summary of behavioral factors of HMIS in health centers of shashogo district Hadiya zone, southern Ethiopia 2021

s.n	Variables			frequency	
	Behavioral factors		yes	no	
		**#**	**%**	**#**	**%**
1	Incentives	208	69.4	92	30.6
2	Knowledge of HMIS	214	71.3	86	28.7
3	Data quality checking skill	168	56	132	44
4	Individual perception motivation	200	66.8	100	33.2
5	Self-efficacy (confidence level)	191	63.65	109	36.5

### Self-efficacy or confidence level of respondents

**Fig. 4:**
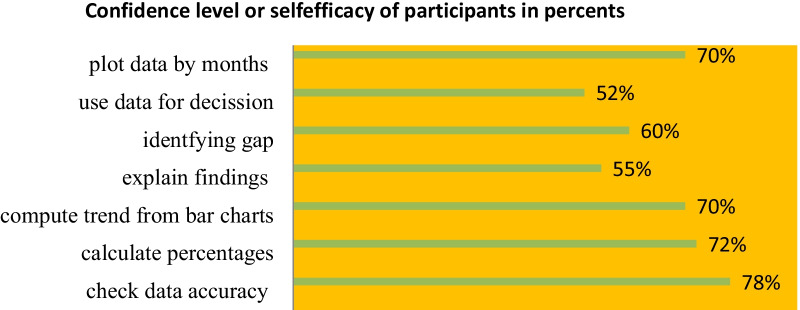
Confidence levels of participants in shashogo district health center 2021

Higher confidence was observed in checking data accuracy at 235 (78%) calculating percentages. 218 (72%) plot the data by month or year, with 210 (70%) identifying gaps by using HMIS data. One hundred eighty (60%) compute trend by bar chart 172 (57%) and lower confidence in using data for making various types of decisions and providing feedback was observed at 158 (52%) relatively. (Fig. 4).

### Organizational factors

Of the total respondents, 180 (60%) reported that they had received training on HMIS activities and another 60 (20%) took pre-service training related to HMIS tasks. (Table 3).

**Table 3:** organizational factors on data quality in shashogo district 2021

SN	Organizational factors	Disagree	agree		
		#	%	#	%
1	Seek feedback from concerned persons	62	20.6	238	79.3
2	Emphasize data quality in monthly reports	61	20.3	239	79.6
3	Discuss conflicts openly to resolve them	59	19.6	241	80.3
4	Seek feedback from concerned community	49	16.3	251	83.7
5	Use HMIS data for setting targets and Monitoring	51	17	249	83
6	Check data quality regularly	50	16	250	84
7	Provide regular feedback to their staff through regular report based on evidence	50	16	250	84
8	Report on data accuracy regularly(Talk to higher level staff about accuracy of data)	50	16	250	84
9	Encourage their supervisees to over report	59	19.6	241	80.3

### Bi variable analysis

Age of respondents, level of education, field of study, years of experience, participation in aggregation or compilation of data, complexity of HMIS formats, registering all activities on a daily basis, filling registration or tally completely, data quality check, supportive supervision, getting feedback, having standard indicators, availability of procedural manuals for data collection, self-efficacy, and registering all activities on a daily basis were identified as candidate variables with *p* < 0.25.

### Multi-variable analysis

Self-efficacy (perceived level of confidence) [AOR = 1.9 95% CI (1.55, 3.35 Respondents with a high level of confidence were 1.9 times more likely to report quality data than those with a low level of confidence.

Supervision [AOR = 3.5 95% CI (1.4, 8.9)], Those supervised healthcare workers were 3.5 times more likely to report quality data or reduce the wrong data compared to those who were not supervised respondents.

Those who completed the registration or formats completely [(AOR = 2.7, 95% CI (1.44, 7.7)], those who completed the registration or formats were 2.7 times more likely to report quality data than those who did not.

Checking data quality [AOR = 1.3, 95% CI (1.5, 3.5)], those health professionals who conduct or check data quality in the health facility were 1.3 times more likely to report quality data compared to those who were not. (Table 4).

**Table 4:** Multi variable logistic regression result on data quality for health centers of shashogo district 2021

S. n	Variable name	Frequency	COR 95%CI	AOR95%CI	*p*-value
1	Checking data quality	Yes 195 (65%)	0.485(0.248,0.948)	1.3(1.5,3.5)	0.041
		No 105 (35%)			
2	Filling registration	Yes 237 (79%)	0.235(0.82,0.678)	2.7(1.44,7.7)	0.035
		No 63 (21%)			
3	Supervision	Yes 280 (93%)	0.570(0.295,1.100)	3.5(1.4,8.9)	0.030
		No 20 (7%)			
4	Self-efficacy	Yes 191 (63%)	1.738(0.949,3.186)	1.9(1.55,3.35)	0.027
		No 109 (37%)			

## Discussion

This study pointed out that the level of data quality in the district was lower than the expected data quality at the national level. Data quality in terms of accuracy, completeness, and timeliness was 79%, 86%, and 84%, respectively. Overall data quality of the district scored 83%, which was below that of the national acceptable level or target of 90% [4]. This might be due to a shortage of skilled human power.

In the current study, the level of data accuracy was 79%, which is almost similar to the study that was conducted in Addis Ababa and was 77.6% [18], and it is higher than a study done in the SNNPR gurage zone that was 32.7%) [19], and the wollega zone data was 48% [20]. This might be due to a difference in the provision of training and computer skills. It is also lower than in Tanzania, where it was 92% [13], and Kenya, where it was 86.7% [21]. This might be due to demographic differences and differences in guidelines from country to country.

The findings of this study showed that the level of content completeness in shashogo district health centers was scored at 86%, which was higher than the study done in Addis Ababa, 33.33% [18], Gomma and Karisa woreda were scored at 75% and 34% [22]. This might be due to differences in worker commitment. It is also higher than studies done in Kenya; the level of data content completeness was 80.5% [21]. This might be due to a difference in time and place of study. It is almost similar to studies done in the East Wollega zone. Oromia regional state content completeness was 86% [20], and in the Gurage zone the result of completeness was reported(87.3%) [19]. The current study was also lower than studies done in Malawi, which was 88% [23] and in Rwanda, the completeness rate was 98% [24]. This is probably due to differences in study periods and sample size.

The overall timeliness in the district health centers was scored at 84% based on a 90% tolerance of timeliness, which was lower than the study done in Addis Ababa city health centers at 96% [18]. This might be due to a difference in the commitment of workers and the coordination of the staff. It is better than studies done in East Wollega zone, Oromia region, which showed 70% [20], Jimma zone, Gomma and Kerisa districts, which showed 70% and 32%, respectively [22] and Kenya, which showed 70.9% [21]. This is probably due to the difference in the number of skilled human power, study period, and sample size determination as well as a difference in study setting in the case of Kenya. The study was nearly similar to a study done in Gombe State, Northeastern Nigeria, at 82% [25].

Checking data quality is important for all health care providers involved in data management. In the current study, approximately 65% of health workers check data quality monthly, which is lower than a study conducted in the Gurage zone, where 95% of participants check data quality monthly [19].This might be due to time and study design differences as well as work overload.

Supervised health workers reported a higher quality of data than those who were not supervised health workers. More than half (66.3%) of the respondents were supervised by their respective higher levels as per standards, and the finding was lower than that of a study done in Kenya where the figure was 79% [26]. This could be due to differences in location and sample size. The current study's findings are higher than those of previous studies in Yaoundé-Cameroon (60%) [27] and Amhara National State 33.4% [28]. This is probably due to a lack of regular supervision from the experts at the top level.

The average confidence level was 63.65%. These findings were almost similar to those conducted in the Gurage zone (66.3%) [19]and Benin (61.4%) [29]. It is higher than studies done in Kenya (57%) [30]and the Amhara region (55.5%) [23]. This variation could be attributed to the fact that the study was conducted in different countries, at different times, and with a different sample size.

According to this study, filling out the registration form completely showed about 79%, which was similar to a study done in the Oromia region, Wollega zone, which found 78.2% [20]. It is higher than a study conducted in Tanzania, 65% [31]. This difference might be due to a sample size difference and the study setting.

## Limitation of the study

Using self-administered questionnaires may be prone to social disability biases and affect the validity of the findings of the study. Content completeness was assessed only for reporting formats, so it couldn't represent the completeness of registration and tally sheets.

## Conclusion

The overall level of data quality in the current study was below the national expected standards. All dimensions of data quality were below 90% in data accuracy, content completeness, and timeliness of data. Conducting supportive supervision, checking accuracy, filling registrations and confidence level were found to be factors associated with data quality. Therefore, all stakeholders should give all necessary support to improve data quality in routine health information systems to truly attain the goal of providing good quality data for the decision-making process by considering the identified factors.

## Data Availability

The datasets generated and/or analyzed during the current study are not publicly available due to the agreement that we have with the sponsored institution not to share the data for third part and there are some irremovable identifier data but are available from the corresponding author on reasonable request.
